# Estradiol modulation of the renin–angiotensin system and the regulation of fear extinction

**DOI:** 10.1038/s41398-019-0374-0

**Published:** 2019-01-29

**Authors:** Jenna N. Parrish, Megan L. Bertholomey, Hong W. Pang, Robert C. Speth, Mary M. Torregrossa

**Affiliations:** 1 Department of Psychiatry, University of Pittsburgh, Pittsburgh, PA 15219 USA; 2 Department of Pharmaceutical Sciences, College of Pharmacy, Nova Southeastern University, Fort Lauderdale, FL 33328 USA; 3 Department of Pharmacology and Physiology, College of Medicine, Georgetown University, Washington, DC 20057 USA

## Abstract

Post-traumatic stress disorder (PTSD) is more prevalent in women than men, yet much remains to be determined regarding the mechanism underlying this sex difference. Clinical and preclinical studies have shown that low estradiol levels during extinction of fear conditioning in rodents (i.e., cue exposure therapy in humans) leads to poor extinction consolidation and increased fear during extinction recall. The renin–angiotensin system (RAS) is also associated with stress-related pathologies, and RAS antagonists can enhance extinction consolidation in males. However, less is known about how estradiol and the RAS converge to alter fear extinction consolidation in females. Since estradiol downregulates the RAS, we determined the role of surgically (via ovariectomy [OVX]) and pharmacologically (via the hormonal contraceptive [HC], levonorgestrel) clamping estradiol at low levels in female rats on fear-related behavior, serum estradiol and angiotensin II (Ang II) levels, and angiotensin II type I receptor (AT1R) binding in the brain. We then tested whether the AT1R antagonist losartan would alter fear-related behavior in an estradiol-dependent manner. We found that both OVX and HC treatment produced extinction consolidation deficits relative to intact female rats in proestrus (when estradiol levels are high), and that losartan treatment mitigated these deficits and reduced freezing. OVX, but not HC, altered AT1R ligand binding, though HC reduced estradiol and increased Ang II levels in plasma. These findings have significant clinical implications, indicating that administration of an AT1R antagonist, especially if estradiol levels are low, prior to an exposure therapy session may improve treatment outcomes in females.

## Introduction

Although women are more than twice as likely to be diagnosed with post-traumatic stress disorder (PTSD) compared with men, few studies focus on understanding mechanisms underlying sex differences in its etiology^1,2^. One core feature of PTSD, persistent re-experiencing of the traumatic event, reflects maladaptive learning and memory processing that has been shown to be influenced by gonadal hormones^2^. Clinical and preclinical research suggests that low estradiol levels during extinction training, where the fear-inducing stimulus is presented in the absence of actual harm, lead to deficits in the consolidation of fear extinction memory, resulting in persistent fear expression during later extinction recall^3–6^. These findings have significant bearing on the efficacy of cue exposure therapy, an extinction-based approach to treating PTSD, in women. However, the precise mechanism by which estradiol regulates extinction consolidation is unknown.

One potential target in understanding how estradiol modulates the consolidation of fear extinction memories is the renin–angiotensin system (RAS), which plays a critical role in modulating sympathetic nervous system tone and cardiovascular health. A retrospective study examining highly traumatized patients found that those who were taking blood pressure medications that act on the RAS had reduced PTSD symptom severity, lower hyperarousal, and decreased intrusive thoughts compared with patients taking blood pressure medications that act independently of the RAS^7^. In addition, male mice treated with an angiotensin II type I receptor (AT1R) antagonist prior to extinction training had decreased fear during extinction recall compared with controls^8^. Thus, drugs that negatively regulate the hypertensive axis of the RAS reduce symptoms of PTSD in human subjects and enhance fear extinction consolidation in males. While there has been some work comparing AT1R radioligand binding between males and females in the periphery^9,10^, the literature is lacking a direct comparison between normotensive male and female AT1R radioligand binding in the brain. Notably, estrogen downregulates the hypertensive axis of the RAS, reducing AT1R expression and enzymes involved in the synthesis of angiotensin II (Ang II), the endogenous ligand for the AT1R^11–13^. Reducing circulating estradiol levels in female rodents via ovariectomy (OVX) significantly increases AT1R binding in both peripheral and brain tissue, which is rescued by estradiol treatment to levels similar to those during proestrus^9,13–16^. Importantly, estrogens regulate angiotensin-converting enzyme and AT1R in female rat anterior pituitary, a region involved in the stress response^17^. The effect of estradiol on Ang II levels is unclear^18–26^, making it possible that estradiol differentially regulates Ang II and AT1R levels. Though estradiol regulation of the RAS has been extensively studied, it is unclear if this interaction affects the consolidation of fear extinction memories, leaving this as an important and open area of investigation that has clear implications for treatment.

Since both estradiol and the RAS are involved in the consolidation of fear extinction memories, we hypothesized that estradiol might promote the consolidation of fear extinction memory through its ability to downregulate the hypertensive arm of the RAS. In the present experiments, we first test whether systemic administration of an AT1R antagonist that is commonly used to treat hypertension (losartan) can reverse the extinction consolidation deficit that is found in female rats with low levels of estradiol, first by OVX, and then by a less invasive and more translationally relevant pharmacological treatment with the hormonal contraceptive (HC), levonorgestrel. Further, the precise mechanism by which estradiol is interacting with the RAS to enhance the consolidation of extinction memory was explored by measuring central AT1R radioligand binding and by determining the concentration of circulating Ang II in serum of female rats with high and low levels of estradiol.

## Materials and methods

### Subjects

Adult female Sprague Dawley rats (Harlan/Envigo, Frederick, MD) were housed 2–3 per cage in individual ventilated cages in a temperature- and humidity-controlled room with food and water available ad libitum. All experimental manipulations were performed during the light cycle (lights on 4:30 a.m.–4:30 p.m.) in accordance with the policies implemented by the University of Pittsburgh Institutional Animal Care and Use Committee.

### Drugs

Levonorgestrel (Sigma-Aldrich), a progestin-only HC shown to downregulate estradiol levels^3^, was dissolved 1:1 in double distilled water and dimethyl sulfoxide (DMSO; Fisher Scientific) and administered subcutaneously (s.c.) at a dose of 0.5 mg/kg/day 4 days prior to and throughout the fear conditioning protocol^3^. Losartan potassium (Sigma-Aldrich), an AT1R antagonist, was dissolved in sterile saline and injected intraperitoneally (i.p.) at doses of 3 mg/kg or 10 mg/kg approximately 5 min before fear extinction^8^.

### OVX and sham surgery

Rats received OVX or sham surgery as described previously^27^. Rats were anesthetized with isoflurane, and a ~2–3 cm incision was made in the abdominal wall. Each oviduct was exteriorized and ligated, and ovaries were excised. The abdominal muscle was closed with silk sutures, and the incision was closed with wound clips. Rats received analgesic (Carprofen, 5 mg/kg) prior to and for 2 days after surgery. Sham surgery rats underwent similar procedures with the exception of excision of the ovaries. Rats were allowed 4 days to recover, and wound clips were removed prior to behavioral testing.

### Apparatus

Behavioral testing occurred in operant conditioning boxes in sound-attenuating chambers (Med Associates, Inc., St. Albans, VT). Boxes were equipped with a shock generator, tone generator, house light, and an exhaust fan. Fear conditioning (Context A) and extinction training/recall testing (Context B) were conducted in chambers with distinctly different walls, flooring, and odors.

### Fear conditioning

On Day 1 (fear conditioning), rats were placed in Context A and were given 3 min to acclimate before presentation of a 10-s, 75 dB tone (conditioned stimulus [CS]), which co-terminated with a 1 mA, 1-s shock, repeated five times with a 1-min inter-trial interval (ITI). On Day 2 (extinction training), rats were placed into Context B and were presented with 30 CSs on a similar schedule as during conditioning, but in the absence of shock. Rats received vaginal lavage (described below) immediately after extinction training. Testing on Day 3 (extinction recall) was identical to Day 2. Time spent freezing (seconds) during each CS, defined as the lack of movement other than breathing, was evaluated by a trained scorer blind to experimental conditions.

### Vaginal cytology

Saline (150 μL) was flushed into the vagina, loaded onto a slide, and coverslipped. Estrous cycle phase on the day of extinction training was determined by cellular morphology (visualized at 200×) according to published protocols^27,28^. Low estradiol states (estrus, metestrus, diestrus) in OVX rats and high estradiol states (proestrus)^3,29^ in sham rats on the extinction training day were confirmed, and only intact rats clearly in proestrus were included in the analysis.

### Enzyme immunoassay

Trunk blood was collected the day after extinction recall. Serum was analyzed for estradiol (Calbiotech Mouse/Rat Estradiol ELISA) and Ang II (Raybiotech Rat ANGII EIA) using commercially available kits according to the manufacturer’s instructions.

### Autoradiography

Brains were flash frozen and stored at −80 °C. Sections (20 μm) were mounted in series of sequential sets of five. AT1R autoradiography was performed as previously described^30^. Briefly, slides were mounted into commercially available slide grips and inverted into Coplin jars containing 35–40 mL of 10 μM PD 123319, an angiotensin II type 2 receptor (AT2R) antagonist with (“nonspecific binding”) or without (“total AT_1_R binding”) 10 µM losartan, in the assay medium buffer for a 30-min pre-incubation period. Slides were then transferred to Coplin jars containing predetermined concentrations of ^125^I-SI-Ang II^30^ along with the respective inhibitors described in the pre-incubation step, and incubated for 60–90 min at room temperature. The concentrations of ^125^I-SI-Ang II were targeted to 200 pM and ranged from 195 to 220 pM. Brains from the different groups were run in parallel to avoid any effects of day-to-day variations in the protocol on the group comparisons. Slides were then quickly rinsed in distilled water, then sequentially rinsed four times in assay medium buffer 1 min each, quickly rinsed in ice-cold distilled water, and dried under a stream of air. Sample slides and at least one ^125^Iodine calibration standard slide were placed in strap-back X-ray cassettes and exposed to Biomax MR-1 X-ray film for several days. The films were then developed and fixed. Slides and films were scanned and Image J was used to perform densitometric analyses of ^125^I-SI Ang II binding to multiple brain regions of interest and the ^125^Iodine standards. Specifically, the anterior piriform cortex (*n* = 70 sections per rat), infralimbic cortex (*n* = 25 sections per rat), basolateral amygdala (*n* = 20 sections per rat), posterior piriform cortex (*n* = 45 sections per rat), paraventricular nucleus of the hypothalamus (PVN) (*n* = 10 sections per rat), ventral subiculum (*n* = 20 sections per rat), and pituitary gland (*n* = 10 sections per rat) were examined. A centered third-order polynomial was used to generate standard curves for the ^125^Iodine standards to convert densitometric readings to fmol/g values. Nonspecific binding was subtracted from total AT1R binding to derive specific AT1R binding, and these values were averaged for each brain region.

### Procedures

**Fig. 1:**
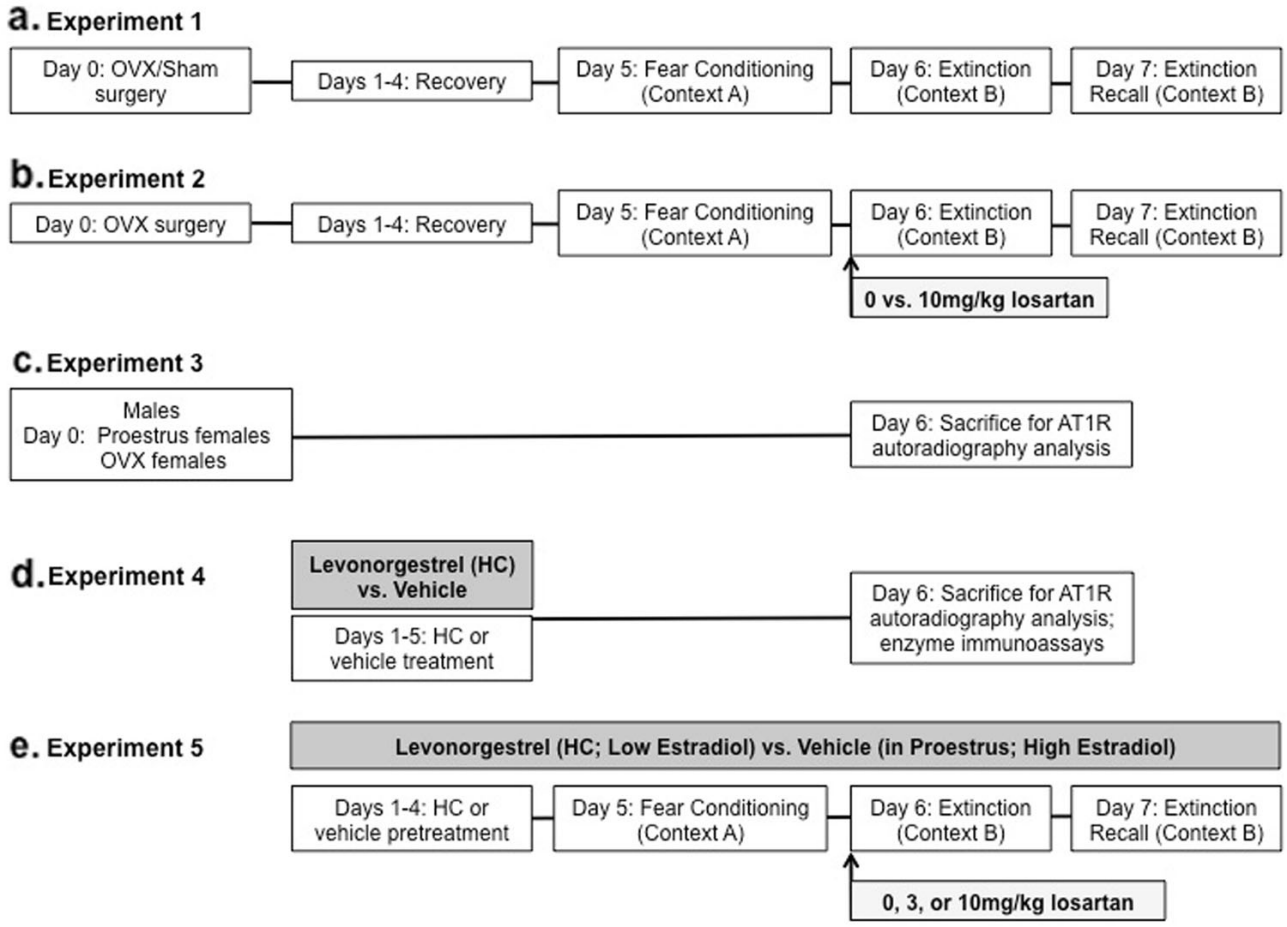
Timeline of experimental procedures. A timeline for Experiments 1–5 (**a**–**e**, respectively) shows each day of experimental manipulations. OVX ovariectomy, AT1R angiotensin II type 1 receptor, ↑ acute losartan treatment, gray shaded area = daily levonorgestrel (hormonal contraceptive/HC) treatment

A timeline for the experimental procedures can be found in Fig. 1. Rats were randomly assigned to their respective treatment conditions at the beginning of each experiment. To determine the effects of circulating estradiol on fear conditioning, rats received either OVX or sham surgery prior to testing in Experiment 1. To evaluate interactions between estradiol and the RAS, OVX females received either losartan (10 mg/kg) or saline 5 min before extinction training in Experiment 2. To further evaluate hormone-related changes in AT1R binding in brain tissue, three groups of rats were used in Experiment 3: males; gonadally intact sham surgery proestrus females; and OVX females. To determine whether a more translationally relevant method of clamping estradiol at a low level, a pharmacological, rather than surgical method was used in Experiments 4–5. In Experiment 4, the effects of HC on serum estradiol, Ang II, and AT1R binding in the brain was determined by injecting levonorgestrel [HC] (vs. control vehicle injections) for 5 days prior to sacrifice via rapid decapitation. Finally, to determine whether HC treatment would produce effects similar to losartan in OVX rats (as in Experiment 2), female rats received either HC or vehicle treatment four days prior to and throughout the fear conditioning paradigm, and were treated with either 0, 3, or 10 mg/kg losartan 5 min prior to the extinction training session in Experiment 5.

### Statistical analysis

Statistical analyses were conducted using IBM SPSS Statistics v 22 (IBM Corporation, Armonk, NY). Tests of normality and homogeneity of variance showed that all data met the assumptions of the general linear model. Sample sizes for each experiment are shown in the figures and/or figure captions, and provide sufficient power based on previous studies. In the behavioral experiments, percent freezing during fear conditioning, extinction training, and extinction recall was analyzed using multifactorial analysis of variances (ANOVAs) with hormone status (high estradiol [sham/intact] vs. low estradiol [OVX; Experiments 1–2]; vehicle vs. HC [Experiment 5] and/or losartan treatment (0, 3, or 10 mg/kg; Experiments 1, 2, and 5) as between-subjects factors and CS or CS block as within subjects factors (pre-CS is the 10 s preceding the first CS and then either the 5 CSs during conditioning or bins of 5 CSs during extinction and extinction recall)). For the autoradiography experiments, pairwise comparisons were made as a function of hormone status. In Experiment 3, because we had a priori hypotheses regarding AT1R ligand binding between intact females vs. males and intact females vs. OVX females, differences in binding were compared via independent samples *t*-tests with planned comparisons. In Experiment 4, vehicle vs. HC groups were compared for AT1R binding and plasma estradiol and Ang II levels by one-tailed independent samples *t*-tests. The significance threshold was set at *p* < 0.05, and *Bonferroni* post-hoc comparisons were performed when appropriate.

## Results

### Impaired fear extinction consolidation in low estradiol OVX females is rescued by systemic losartan treatment

**Fig. 2:**
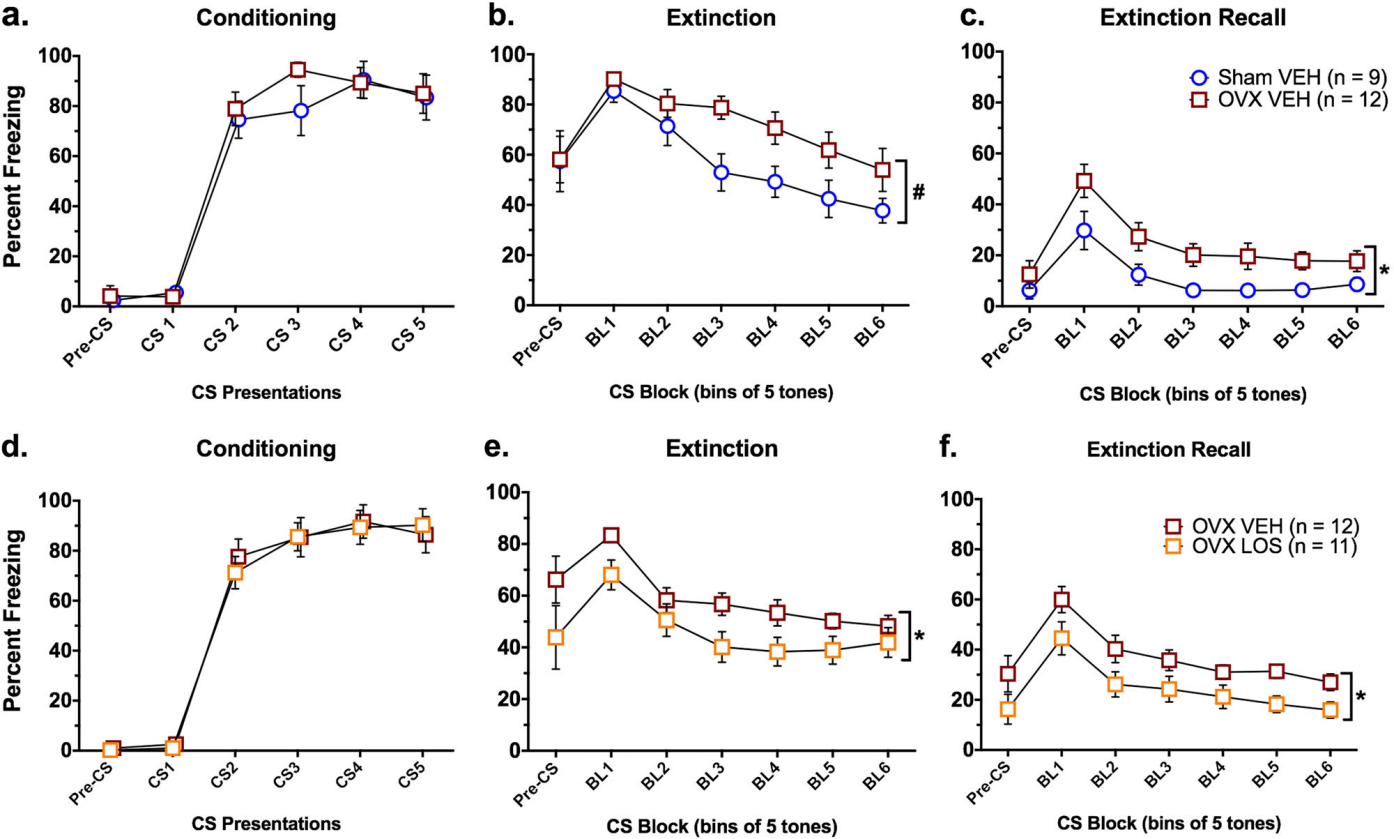
Losartan rescues extinction consolidation impairment in OVX females. **a** No group differences in freezing were evident during fear conditioning in ovariectomized (OVX) or gonadally intact sham-operated (Sham) rats. **b** A trend for increased freezing was found in OVX females during extinction training compared with Sham proestrus females. **c** OVX females exhibited significantly greater freezing compared with sham females during extinction recall, indicating that extinction consolidation is disrupted in the OVX females. **d** No group differences in freezing were evident during fear conditioning in the two groups of OVX rats. **e** OVX females treated with 10 mg/kg losartan spent significantly less time freezing during the extinction session compared with vehicle-treated females; however, no significant differences in freezing were observed between groups during the last CS block. **f** Freezing was significantly reduced in OVX losartan-treated females during extinction recall compared with the OVX vehicle-treated group. *p < 0.05; ^#^*p* < 0.10

In Experiment 1, we replicated the findings of Milad and colleagues^3–6,31,32^ of impaired fear extinction consolidation in low estradiol OVX females compared with sham-operated, high estradiol, proestrus females. Consistent with other studies, OVX had no effect on fear conditioning (Fig. 2a), with a significant main effect of CS number [*F*(4, 76) = 75.0, *p* < 0.001], but no effect of surgery or interaction, indicating that both groups acquired similar levels of conditioned fear. In contrast, during extinction training, a main effect of surgery neared significance (*p* = 0.05), with OVX females exhibiting higher levels of freezing relative to shams (Fig. 2b). A significant main effect of CS block [*F*(5, 95) = 36.75, *p* < 0.001] revealed that freezing during blocks 1–4 was significantly higher than blocks 5–6, indicative of extinction of conditioned fear in both groups. However, a significant CS block × surgery interaction [*F*(5, 95) = 2.39, *p* = 0.04] showed that freezing was significantly higher in OVX females compared with sham females for blocks 3 and 4, suggesting impaired extinction learning in rats with low estradiol levels. Similarly, a significant main effect of surgery was found during extinction recall [*F*(1, 19) = 6.32, *p* = 0.02], where OVX females exhibited higher levels of freezing compared with shams (Fig. 2c), similar to recent findings^33^. A significant main effect of CS block [*F*(5, 95) = 26.03, *p* < 0.001] showed that freezing in block 1 was significantly higher than all subsequent blocks.

Experiment 2 tested the hypothesis that systemic treatment with the AT1R antagonist losartan would enhance fear extinction consolidation in OVX females. All rats increased freezing across CS presentations during conditioning [*F*(4, 84) = 79.97 *p* < 0.001; (Fig. 2d)]. During extinction training, a significant main effect of treatment [*F*(1, 21) = 5.79, *p* = 0.03] indicated that losartan reduced freezing in OVX females compared with vehicle-treated controls (Fig. 2e). These effects were most evident at the beginning of the extinction session, as no group differences were observed by the end of extinction training. An overall main effect of CS block [*F*(5, 105) = 20.67, *p* < 0.001] showed that freezing during the first block was significantly higher than all subsequent blocks. Similar findings were evident during extinction recall, wherein losartan-treated females exhibited significantly less freezing compared with vehicle-treated females ([*F*(1, 21) = 6.22, *p* = 0.02]; Fig. 2f), with overall decreases in freezing after the first CS presentation [*F*(5, 105) = 28.47, *p* < 0.001]. Thus, blockade of AT1R was able to rescue deficits in fear extinction consolidation induced by removal of the primary source of estradiol in female rats.

### AT1R radioligand binding in the pituitary gland and ventral subiculum is negatively regulated by estradiol

**Fig. 3:**
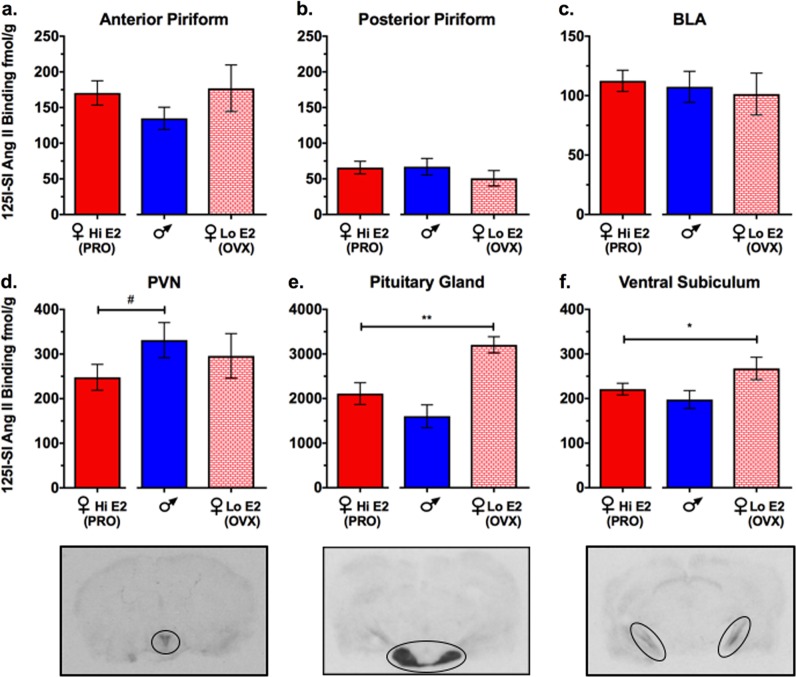
Ovariectomy (OVX) females have significantly higher angiotensin II type I receptor (AT1R) ligand binding in the anterior pituitary gland and ventral subiculum compared with intact proestrus females. No significant differences were found between intact proestrus females (♀HI E2; PRO) and any other treatment group (*n* = 7–17/group) in the **a** anterior piriform cortex, **b** posterior piriform cortex, or **c** basolateral amygdala (BLA). **d** Males (♂) had a trend for increased AT1R binding in the PVN compared with intact PRO females. **e** OVX females had significantly higher AT1R ligand binding in the anterior pituitary gland and **f** ventral subiculum compared with intact PRO females. Representative autoradiograms from a high estradiol proestrus female can be found below Figs. 2d–f. From left to right, encircled areas are paraventricular nucleus of the hypothalamus, anterior pituitary and ventral subiculum. **p < 0.01; *p < 0.05; ^#^*p* < 0.10

We next tested whether regulation of the RAS was estradiol dose dependent by comparing intact proestrus females with OVX females and males in Experiment 3. Planned comparisons failed to detect significant differences between groups in the anterior piriform cortex, posterior piriform cortex, and basolateral amygdala (BLA) (Figs. 3a–c, respectively). There was a trend for increased AT1R binding in the PVN of males compared with intact proestrus females (*p* = 0.06; Fig. 3d). OVX females had significantly higher AT1R binding in the anterior pituitary gland [*t*(19) = 2.93, *p* = 0.004] and ventral subiculum [*t*(22) = 1.80, *p* = 0.04] compared with intact proestrus females (Figs. 3e and f, respectively), suggesting that estradiol downregulates AT1R levels in these regions.

### HC treatment reduces estradiol and increases Ang II but does not alter AT1R radioligand binding

**Fig. 4:**
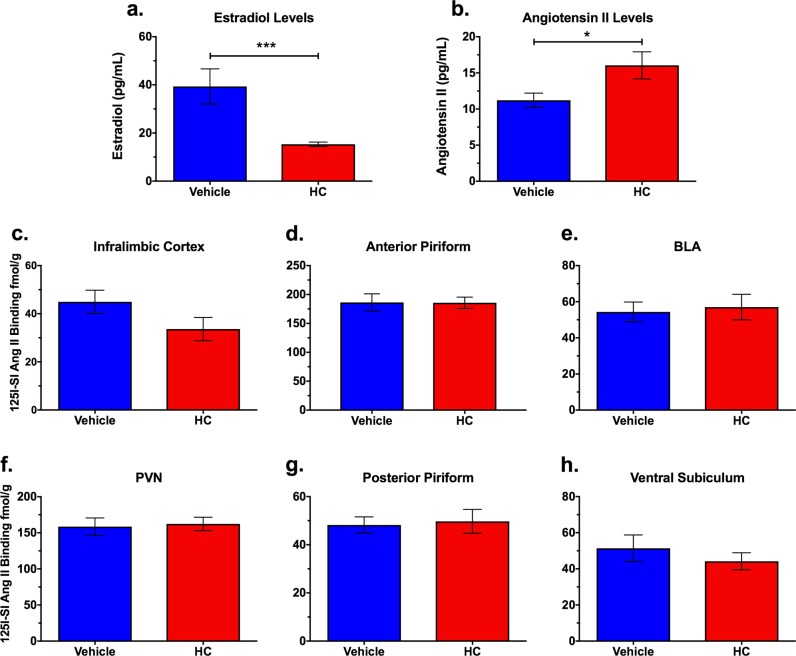
Hormonal contraceptive (HC)-treated females with low estradiol levels have significantly elevated Ang II levels, but did not affect angiotensin II type I receptor (AT1R) binding. **a** Estradiol levels are significantly reduced in HC-treated females compared with proestrus vehicle-treated intact females (*n* = 7–12/group). **b** HC-treated females (low E2) have significantly elevated Ang II levels compared with vehicle-treated proestrus females (high E2). No significant differences in AT1R ligand binding were detected between HC- and vehicle-treated gonadally intact female rats in the **c** infralimbic cortex, **d** anterior piriform cortex, **e** basolateral amygdala (BLA), **f** paraventricular nucleus of the hypothalamus, **g** posterior piriform cortex, or **h** ventral subiculum. ***p < 0.001; *p < 0.05

To further elucidate the mechanism by which estradiol regulation of the RAS alters extinction consolidation, in Experiment 4 we first verified that HC treatment significantly reduced circulating estradiol levels compared with vehicle-treated proestrus females [*t*(18) = 4.01, *p* < 0.001] (Fig. 4a). HC treatment also significantly elevated circulating Ang II levels, [*t*(20) = 1.86, *p* = 0.04] (Fig. 4b). However, despite indications that estradiol affects AT1R levels through a post-transcriptional mechanism^14,34^, no significant differences in AT1R ligand binding were found between HC-treated females and vehicle-treated proestrus females for any region examined (all *p* > 0.05; Figs. 4c–h).

### Impaired fear extinction consolidation in low estradiol HC-treated females is rescued by systemic losartan treatment

**Fig. 5:**
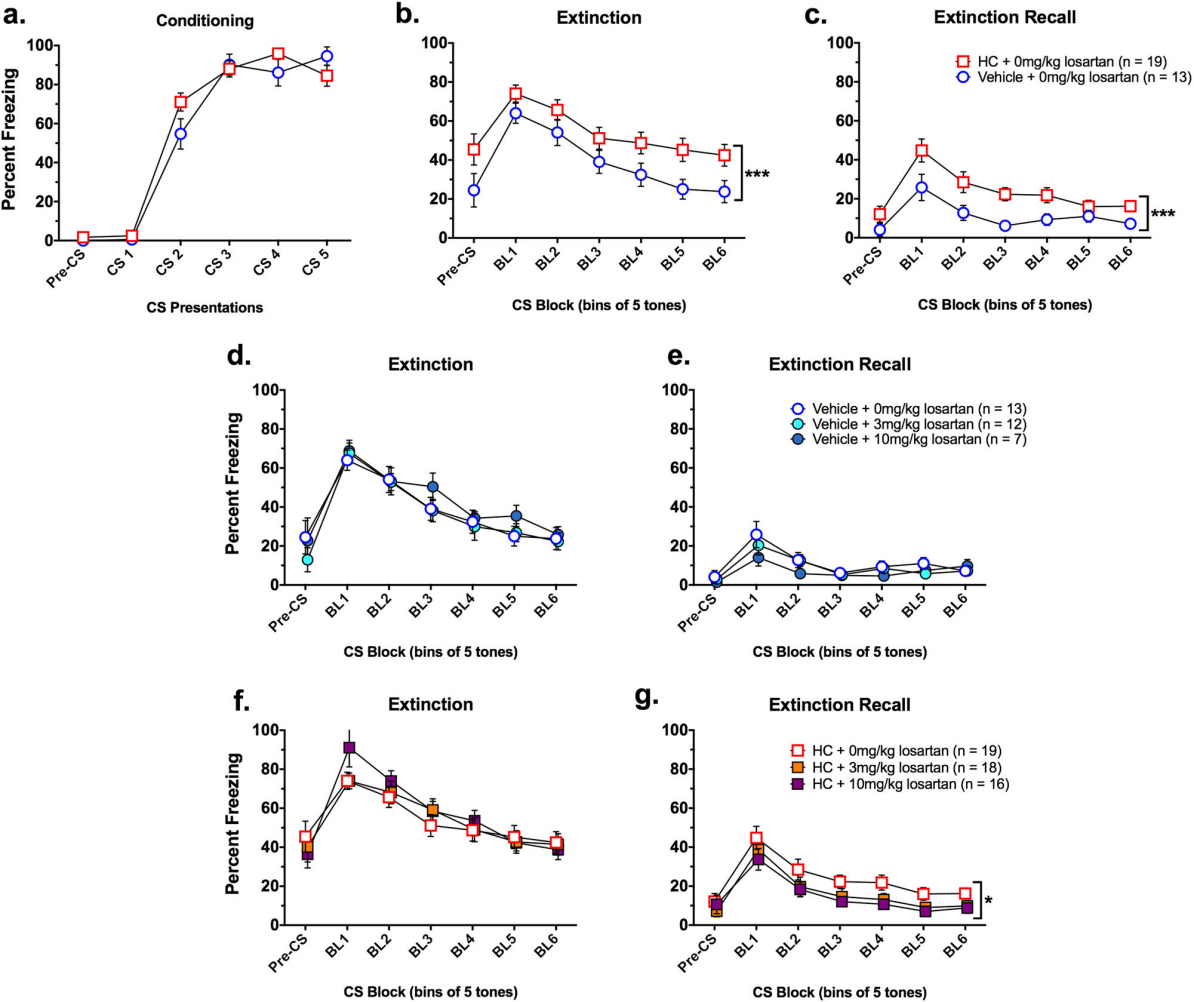
Female rats treated with hormonal contraceptive (HC) exhibit impaired extinction consolidation, which can be rescued with systemic losartan. Mean (±SEM) freezing levels are shown across the fear conditioning, extinction, and extinction recall sessions. **a** No significant differences in freezing were found between HC- and vehicle-treated gonadally intact females during fear conditioning. **b** A main effect of HC treatment was found during extinction, where HC-treated females had significantly higher freezing levels compared with vehicle-treated controls. **c** HC-treated females displayed significantly higher levels of freezing compared with vehicle-treated control females during extinction recall. **d** Losartan treatment alone had no effect on freezing during extinction in high estradiol vehicle-treated control females. **e** Losartan treatment alone had no effect on freezing during extinction recall in high estradiol vehicle-treated control females. **f** Losartan treatment did not affect freezing levels during extinction in HC-treated females. **g** The 10 mg/kg dose of losartan significantly reduced freezing during extinction recall in HC-treated females compared with HC-treated females in the 0 mg/kg losartan group. ***p = 0.001; *p < 0.05

To implement a more treatment-based approach, we replicated Experiment 2 using HC, rather than OVX, to reduce estradiol levels in Experiment 5. As in Experiments 1–2, estradiol levels had no effect during fear conditioning (*p* > 0.05; Fig. 5a), and rats acquired conditioned fear (main effect of CS presentation; [*F*(4, 132) = 166.43, *p* < 0.001]). A significant CS × treatment interaction was found, but appeared to be due to non-systematic (i.e., not following any predictable pattern) variability between the HC- and vehicle-treated groups across time.

During extinction training, a main effect of hormone treatment [*F*(1, 79) = 11.95, *p* = 0.001] showed reduced freezing in vehicle-treated compared with HC-treated rats (Fig. 5b), consistent with other studies using HC^3^ and the findings from Experiment 1 in OVX rats. A main effect of CS block [*F*(5, 395) = 137.16, *p* < 0.001] similarly showed that the highest and lowest levels of freezing were in CS Block 1 and CS Block 6, respectively, demonstrating sufficient extinction acquisition. No significant effects of losartan were found during extinction training in vehicle-treated (Fig. 5d) or HC-treated (Fig. 5f) females, indicating that losartan does not affect extinction acquisition, similar to what has been found in males^8^.

During extinction recall, a main effect of CS Block [*F*(5, 395) = 50.96, *p* < 0.001] confirmed decreases in freezing across the session. A main effect of HC treatment [*F*(1, 79) = 11.05, *p* = 0.001] and a CS block × HC treatment interaction [*F*(5, 395) = 7.75, *p* < 0.001] revealed that the HC-treated groups had higher levels of freezing compared with vehicle-treated groups across CS Blocks 1–4 (Fig. 5c). We then tested the effect of 3 and 10 mg/kg losartan on extinction recall in both the vehicle and HC treatment groups. The 3 mg/kg dose was based on a previous study showing enhanced extinction consolidation in male mice^8^, whereas the 10 mg/kg dose was selected based on the hypothesis that a higher dose of losartan may be necessary to overcome the HC deficit. Subsequent ANOVAs revealed that losartan treatment had no significant effect on freezing in the extinction recall test in the vehicle-treated proestrus rats (Fig. 5e; *p* > 0.05), which was expected, given that these rats already exhibited very low levels of freezing. However, there was a trend for losartan treatment to dose dependently reduce freezing in the HC-treated groups (*p* = 0.10). Exploratory 2 × 6 (treatment by CS block) ANOVAs failed to detect any differences between the 3 mg/kg losartan dose and any other dose tested. However, consistent with the hypothesis that larger losartan doses would be needed to surmount the HC-related extinction consolidation deficit, we found that the 10 mg/kg dose of losartan normalized freezing in HC-treated females, evidenced by significant decreases in freezing compared with the 0 mg/kg losartan group [*F*(1, 33) = 4.45, *p* = 0.04] (Fig. 5g).

## Discussion

The results of the present experiments suggest that the deficit in extinction consolidation, which is observed in females with surgically (via OVX) or pharmacologically (via HC treatment)-induced low estradiol levels, can be rescued by systemic treatment with the AT1R antagonist losartan prior to extinction training. We also found that AT1R ligand binding in OVX females is elevated in the anterior pituitary gland (consistent with previous studies^17^) and ventral subiculum, both of which are known to affect hypothalamic-pituitary-adrenal (HPA) axis activity. Although no significant differences were found between HC- and vehicle-treated females in AT1R ligand binding, levels of the Ang II peptide were significantly elevated in HC treated, low estradiol females compared with high estradiol females. Thus, our findings suggest that increased Ang II signaling at either the peptide or receptor level may contribute to fear extinction deficits in low estradiol females, and that AT1R antagonists may be useful adjuncts to exposure therapy in specific patient populations.

Consistent with previous studies, we found that OVX and HC-treated females had deficits in extinction consolidation^3,33^. Similar to other studies^3^, we also found a significant main effect of HC treatment during extinction training, where HC-treated females had significantly increased freezing levels compared with vehicle-treated proestrus females. We found that freezing levels between HC- and vehicle-treated females were similar at the beginning of the extinction training session and differ towards the end of extinction, which suggests that HC-treated females may have some resistance to extinction, rather than stronger consolidation of the initial fear memory. Importantly, we are the first to find that OVX and HC-treated females that receive 10 mg/kg losartan prior to extinction training have improved extinction consolidation and reduced freezing during extinction recall. No effect of losartan was found in proestrus (high estradiol) females, likely because extinction learning was optimal in these rats, resulting in extremely low levels of freezing during recall. Although we also found a significant main effect of losartan treatment during extinction training in OVX females, these differences were most prevalent during the beginning of the extinction session before losartan was likely exerting pharmacological effects^8,35^, suggesting that other factors might be contributing to these effects. Further, no effects of losartan were evident during extinction training in Experiment 4 (Figs. 5d, f). Importantly, freezing during the extinction session did not differ by the final block of extinction, allowing us to compare fear levels between these two groups during the extinction recall session. Although we did not directly test how this dose of losartan affects anxiety-like behavior and locomotor activity, findings in these experiments are similar to a previously published study showing that systemic losartan given prior to extinction training enhances extinction consolidation in male mice without affecting baseline anxiety, blood pressure, and distance traveled in an open field^8^. While other studies in normotensive male rats have shown that a 10 mg/kg dose of losartan affects blood pressure 6 h after a single treatment, blood pressure is normalized 24-h post-injection^35^. Thus, in our experiments, losartan is most likely not on board during extinction recall, and its effects on freezing during extinction recall can be attributed to its effect during the extinction consolidation window^36^. Our findings suggest that losartan, which is commonly prescribed for the treatment of hypertension, can rescue deficits in extinction consolidation that may occur in patients undergoing exposure therapy in a low estradiol state. Enhanced extinction learning should improve treatment outcome for patients with anxiety disorders such as PTSD.

Recent studies have shown that elevated HPA axis activity is associated with poor extinction consolidation in a rodent model of PTSD, and that dampening HPA axis activity in these rodents enhances extinction consolidation^37,38^. Interestingly, the main findings of our AT1R autoradiography studies revealed that OVX females had significantly elevated AT1R ligand binding in the pituitary gland and ventral subiculum, regions that affect HPA axis activity. In the pituitary gland, AT1Rs are primarily found in the anterior rather than posterior pituitary gland^39^, and the anterior pituitary gland releases ACTH in response to stressors. While in vitro studies suggest that Ang II can directly stimulate release of ACTH from pituitary cells^40^, in vivo studies suggest that Ang II most likely mediates the increase in ACTH through its actions in the circumventricular organs^41^, though further study is needed. Interestingly, we also found that AT1R ligand binding in OVX females was increased in the ventral subiculum^42^. Lesions of the ventral subiculum have been found to enhance corticotrophin releasing factor mRNA and peptide expression in the PVN and elevate corticosterone levels following restraint stress^43,44^. Although the role of angiotensin signaling in the ventral subiculum is currently unknown, it is possible that Ang II signaling in this region could interfere with ventral subiculum-mediated inhibition of the HPA axis, which would lead to increased HPA axis activity and poor extinction consolidation.

During extinction training, a time in which stress responses are high, we propose that Ang II, which is increased both in the brain and periphery in response to stressors^45,46^, binds to AT1Rs in the pituitary gland and ventral subiculum, which results in increased HPA axis activity, and poor extinction consolidation in OVX females. Conversely, blockade of AT1R by losartan reduces Ang II-mediated increases in ACTH (adrenocorticotropic hormone) in normotensive men^47^, suggesting a stress-dampening effect (though studies in women are needed). Thus, we hypothesize that treatment with losartan prior to the extinction training session in OVX females blocks AT1Rs in these regions, blunting the neuroendocrine response to stress, thereby enhancing extinction consolidation and reducing fear during extinction recall, although further testing is needed to validate our hypothesis. Future studies will explore how components of the HPA axis (i.e. CRH, ACTH, corticosterone) are affected in OVX vehicle-treated and OVX losartan-treated females during the extinction consolidation window, and whether AT1R blockade in the pituitary gland or ventral subiculum reverses the deficit in extinction consolidation in OVX females. Further, in an effort to inform long-term therapies, future studies should also test the degree to which manipulations of ovarian hormones and/or the RAS alter fear extinction retention to lead to persistent reductions in the expression of fear responses.

Unlike our findings in OVX females, AT1R radioligand binding was not altered in HC-treated females. One possible mechanism for this is that OVX leads to decreased levels of both estrogen and progesterone, the latter of which also has been shown to decrease AT1R binding^48,49^; however, since levonorgestrel also reduces levels of both ovarian hormones, progesterone is not likely playing a role in the differential effects of HC and OVX on AT1R binding. Rather, these two treatments are likely modulating the RAS through different mechanisms, possibly in a hormone dose-dependent manner. For example, although OVX increased AT1R levels, HC treatment significantly increased Ang II peptide levels. This finding is consistent with some studies, but not others. For example, one study found no significant differences in plasma or cortical Ang II levels between male and female Lewis rats^20^. Similarly, no significant differences in plasma Ang II were found between men and women in their early twenties; however, males had significantly elevated Ang 1–7 compared with females^26^. Surprisingly, one study examining plasma Ang II levels in OVX female rats found that they had significantly reduced plasma Ang II, and treatment with estradiol reversed this effect^18^. Although OVX in rats is not necessarily a model of menopause, parallel findings do show that postmenopausal women taking an estrogen supplement have reduced plasma Ang II levels^24^. Finally, women taking HCs that contain both estrogen and progesterone have significantly elevated levels of plasma Ang II compared with healthy females that were not taking HCs and males^19,23^, similar to our results reported here. Since findings on circulating Ang II levels are inconsistent across studies, broad conclusions are difficult to draw, but our results suggest similar effects of HC treatment in rats as in humans that may be relevant for the efficacy of exposure-based therapies. We acknowledge that the methodology used for measuring Ang II peptide levels in this study can detect other angiotensin peptides; therefore, we cannot rule out that some behavioral effects could be mediated by these peptides rather than or in addition to Ang II. Nevertheless, the AT1R antagonist was effective in improving extinction recall in HC-treated rats, suggesting that these animals have increased brain AT1R signaling by some mechanism.

In summary, we are the first to show that systemic treatment with AT1R antagonist losartan prior to extinction training results in significant improvements in fear extinction consolidation in both OVX and HC-treated females. Therefore, treatment with losartan, which has been previously shown to reduce PTSD symptoms in highly traumatized patients with hypertension and increase extinction consolidation in male mice^7,8^, or treatment with other AT1R antagonists, may provide superior treatment benefits in women with low levels of estradiol undergoing therapy for PTSD. The enhancements in extinction consolidation may reduce responses to fear-related cues outside of the treatment clinic. While it would not be harmful for patients with high estradiol levels to take an AT1R antagonist prior to exposure therapy, our results suggest that this treatment may have limited benefits for these patients. Recent studies provide genetic evidence that some individuals may respond better to AT1R antagonists as treatment for PTSD^50^; and our results support the idea that specific genetic and hormonal factors can influence treatment efficacy. Thus, individualized treatment plans may be required for the successful treatment of many psychiatric disorders, as sex, genetic predispositions, and hormonal state are important factors that need to be taken into account when developing treatment plans.
